# Effects of Dietary Strawberry Supplementation on Antioxidant Biomarkers in Obese Adults with Above Optimal Serum Lipids

**DOI:** 10.1155/2016/3910630

**Published:** 2016-06-27

**Authors:** Arpita Basu, Stacy Morris, Angel Nguyen, Nancy M. Betts, Dongxu Fu, Timothy J. Lyons

**Affiliations:** ^1^Department of Nutritional Sciences, College of Human Sciences, Oklahoma State University, Stillwater, OK 74078, USA; ^2^Centre for Experimental Medicine, Queen's University of Belfast, Belfast BT7 1NN, UK; ^3^Section of Diabetes & Endocrinology, University of Oklahoma Health Sciences Center, Oklahoma City, OK 73104, USA

## Abstract

Berries have shown several cardiovascular health benefits and have been associated with antioxidant functions in experimental models. Clinical studies are limited. We examined the antioxidant effects of freeze-dried strawberries (FDS) in adults [*n* = 60; age: 49 ± 10 years; BMI: 36 ± 5 kg/m^2^ (mean ± SD)] with abdominal adiposity and elevated serum lipids. Participants were randomized to one of the following arms: low dose strawberry (25 g/day FDS), low dose control beverage (LD-C), high dose strawberry (50 g/d FDS), and high dose control beverage (HD-C) for 12 weeks. Control beverages were matched for calories and total fiber. Plasma antioxidant capacity, trace elements (copper, iron, selenium, and zinc), whole blood glutathione (GSH), and enzyme activity (catalase, glutathione peroxidase, and glutathione reductase) were examined at screening (0 week) and after 12 weeks' intervention. At 12 weeks, plasma antioxidant capacity and glutathione levels were higher in the strawberry versus control groups (low and high dose FDS: 45% and 42% for plasma antioxidant capacity and 28% and 36% for glutathione, resp.); glutathione was higher in the high versus low dose strawberry group (all *p* < 0.05). Serum catalase activity was higher in the low dose strawberry (43%) versus control group (*p* < 0.01). No differences were noted in plasma trace elements and glutathione enzyme activity. Dietary strawberries may selectively increase plasma antioxidant biomarkers in obese adults with elevated lipids.

## 1. Introduction

Dietary berries, a rich source of bioactive compounds, especially the polyphenols, have been associated with protective effects against chronic diseases, especially lowering risk factors of cardiovascular disease (CVD) [1–3]. Oxidative stress is one of the major contributors to the pathophysiology of CVD and is triggered by many factors, such as obesity, elevated serum lipids, and low dietary and cellular antioxidant status [4, 5]. The antioxidant system is a complex network of various biomolecules that individually and synergistically counteract free radicals and protect against oxidative damage. The antioxidant enzymes, such as catalase and the superoxide dismutase (SOD), serve as first line defenses against hydrogen peroxide and superoxide anions, respectively, while the glutathione enzyme system counteracts the peroxide molecules generated as by-products of dismutase activity, and from the oxidation of biomolecules that results in lipid peroxides [4]. In addition to these antioxidant enzymes, dietary micronutrients, such as vitamins C and E, and trace elements, such as copper, iron, selenium and zinc, also play an important role in antioxidant and pro-oxidant activities [6]. Many polyphenol-containing foods and beverages, such as berries, cocoa, and green tea, as well as their extracts, have been reported to enhance the activities of antioxidant enzymes and to chelate metal ions, thereby improving oxidative stress [7–9]. However, few clinical studies have examined their effects in adults with cardiovascular risk factors, such as those with obesity, dyslipidemia, and the metabolic syndrome.

Among the popular sources of dietary berries, strawberries have been shown to alleviate CVD risk factors in clinical studies as well as in animal models of atherosclerosis [10]. However, only a few studies have reported their effects on antioxidant biomarkers, especially on antioxidant enzyme functions such as catalase and the glutathione enzyme system. Using an animal model of oxidative stress, pretreatment with strawberries was shown to attenuate the ethanol-induced decreases in catalase and SOD activities in the gastric mucosa of rats [11]. Similar findings have been reported from other studies using green tea and fruit extracts [12, 13]. Clinical trials mostly in studies of short duration in healthy volunteers have shown the effects of different forms of dietary berries in increasing plasma antioxidant capacity and thiol groups, but no effects on glutathione enzymes [14]. In a previously reported eight-week study of green tea supplementation in adults with the metabolic syndrome, we reported a significant increase in whole blood glutathione and a decrease in plasma iron as a result of green tea intervention [15]. Emerging research supports the role of berries, such as raspberries and raspberry seed oil, and native fruit extracts such as the* Grewia asiatica* fruit in increasing the intracellular concentrations of glutathione and in modulating antioxidant enzyme activities in experimental models of oxidative stress and dyslipidemia [16–18]. While these study findings are interesting, the practical relevance is limited based on the dosing, as well as the use of test agents that are part of complementary or folk medicine and not commonly consumed in the daily human diet.

Thus, there is a need to investigate the effects of berries on antioxidant biomarkers in clinical studies using achievable dietary doses. In a dose-response study, our group previously reported the effects of dietary freeze-dried strawberries in lowering total and LDL-cholesterol and lipid peroxidation in adults with abdominal obesity and elevated serum lipids [19]. Using stored samples from our previous study [19], we measured selected biomarkers of antioxidant status following low and high dose strawberry intervention. In the current report, we hypothesize that dietary strawberries will improve selected biomarkers of plasma antioxidant status, thus potentially benefitting cardiovascular health, in adults following a 12-week supplementation of two doses of freeze-dried strawberry beverages compared to the calorie and fiber-matched controls. Thus, our specific study objectives were to examine the effects of strawberries on circulating antioxidant biomarkers using stored samples, specifically plasma antioxidant capacity, plasma catalase, glutathione peroxidase and glutathione reductase enzyme activities, whole blood glutathione concentrations, and plasma trace elements in obese adults with above normal serum lipids.

## 2. Methods

The study design has been previously published [19]. Briefly, this was a randomized controlled trial involving low and high doses of freeze-dried strawberries and corresponding calorie- and fiber-matched controls. The study was approved by the Institutional Review Board at the University of Oklahoma Health Sciences Center and at Oklahoma State University. All participants provided written informed consent. The trial is registered with ClinicalTrials.gov supported by the US National Library of Medicine at the National Institutes of Health (https://clinicaltrials.gov/show/NCT01883401).

### 2.1. Participants

Inclusion and exclusion criteria have been described previously [19]. Men and women [aged 49 ± 10 y (means ± SDs)] with abdominal adiposity and elevated serum lipids and who were free of any chronic diseases were enrolled in this randomized controlled study. Recruitment and study procedures were conducted at the Clinical Research Center at the University of Oklahoma Health Sciences Center and at the Nutritional Sciences Clinical Assessment Unit at Oklahoma State University. Participants were recruited using flyers and campus e-mail advertisements at both sites. Each potential recruit received an initial telephone screening before the screening visit.

### 2.2. Interventions

Table 1 describes the composition of the freeze-dried strawberry beverages and the fiber and calorie-matched control beverages used in the study. The freeze-dried strawberries were kindly donated by the California Strawberry Commission (CSC), and the mixture of strawberries used to generate the powder contained the University of California public cultivars as follows: Camarosa (37%), Ventana (13%), Diamante (13%), and 2 proprietary varieties (37%) in production in 2010. Participants were randomly assigned to consume one of the following four beverages for 12 wk: (1) low dose FDS [LD-FDS; 25 g reconstituted in 2 cups (474 mL) of water]; (2) low dose calorie- and fiber-matched control [LD-C; 4 g of fiber and 5 teaspoons (20 g) of cane sugar, blended in 2 cups (474 mL) of water]; (3) high dose FDS [HD-FDS; 50 g reconstituted in 2 cups (474 mL) of water]; or (4) high dose calorie- and fiber-matched control [HD-C; 8 g of fiber and 9 teaspoons (36 g) of cane sugar, blended in 2 cups (474 mL) of water]. The fiber used in control beverages was composed of vegetable fibers and natural gums and contained both insoluble and soluble fiber (1 : 2) per serving (Fiberstir). In addition, the control beverages contained added red food color (McCormick & Company, Baltimore MD) and artificial strawberry-flavored Kool-Aid (Kraft Foods, Pittsburgh PA) to mimic the color and flavor of the FDS beverages. Participants were asked to consume one cup in the morning and the second in the evening, at least 6–8 h apart. Participants were instructed to add the strawberry or control beverages as a snack to their usual diet, and not to use it to replace any meals. Since the strawberry powder is sticky in consistency, especially when reconstituted in water, the participants were provided specific instructions on the preparation and storage of the test drinks and demonstration on how to rinse the cups to ensure no wastage of the test powder. Compliance was assessed by the return of unused test powder and mandatory three days per week visit to the clinic for monitored consumption of the beverages. In addition, plasma ellagic acid was also measured as described previously [19].

### 2.3. Anthropometrics and Clinical and Dietary Analyses

Body weight was recorded on an uncarpeted surface with the SECA 644 Multifunctional Hand Rail Scale (SECA) and recorded to the nearest 0.1 kg. Height was measured without shoes using the Accustat Genentech Stadiometer and recorded to the nearest 0.1 cm. Waist circumference was measured at the superior iliac crest using the Gulick II Tape Measure (Vital Signs). Fasting blood samples were collected, and serum was promptly transported to the University of Oklahoma Medical Center Laboratory for analyses of glucose, lipid profiles (total cholesterol, TG, LDL-cholesterol, and HDL-cholesterol), and other blood variables, including safety variables (hemoglobin, platelets, white blood cells, liver enzymes, creatinine, and blood urea nitrogen) using automated diagnostic equipment (Abbott Architect Instruments) following standard protocols at the University of Oklahoma Medical Center. Micronutrient intakes were estimated based on three-day food records collected at screening and 12 wk of the study. Three-day averages (two weekdays and one weekend day) of micronutrient and fruit and vegetable intakes were analyzed using Nutritionist Pro (version 3.2, 2007; Axxya Systems).

### 2.4. Plasma Antioxidant Capacity and Whole Blood Glutathione

Plasma antioxidant capacity was measured using the assay developed by Miller et al. [20]. The average intra-assay CV was 4.6%. Reduced glutathione content in heparinized whole blood sample was measured using the method described by Beutler et al. [21]. Briefly, 100 *μ*L of hemolyzed blood sample and 200 *μ*L of 2.5 mmol/L 5,5′-dithiobis-2-nitrobenzoic acid (Sigma, St. Louis, Missouri) were mixed in tubes containing 1.9 mL Tris-HCl buffer (pH 8.0). The absorbance of the yellow thiolate anion was measured at 412 nm. Reduced glutathione (Sigma) was used as a standard. Calibration curve was used to calculate concentration and was expressed as micrograms per gram hemoglobin. The average interassay CV was 5.2%.

### 2.5. Serum Catalase, Glutathione Peroxidase, and Glutathione Reductase Activity

Serum catalase activity and glutathione reductase activity were measured using Catalase Assay Kit and the Glutathione Reductase Assay Kit (Cayman Chemical Company, Ann Arbor, Michigan, USA) using the spectrophotometric assays based on the manufacturer's protocol. The average interassay CV was 4.6% and 5.2%, respectively. Glutathione peroxidase was measured by using GPx-340 (OxisResearch) based on the manufacturer's protocol. The average interassay CV was 6.6%.

### 2.6. Trace Element Analysis

Plasma levels of copper, iron, selenium, and zinc were measured using inductively coupled plasma quadrupole mass spectroscopy (Elan 9000; Perkin Elmer, Norwalk, CT) as described [22]. All plasma samples were diluted 20-fold (200 *μ*L diluted to 4 mL) with 0.1% nitric oxide (GFS Chemicals, Powell, OH) in ultrapure water. Standard solutions of selected trace elements were prepared by dilution of certified standard solutions (Perkin Elmer, Norwalk, CT). The calibration standards were prepared in 0.1% nitric acid solution at 0, 50, and 100 *μ*g/L. All samples and standards were spiked with 10 *μ*g/L gallium as an internal standard (Perkin Elmer, Norwalk, CT). Polypropylene plasticware (Sarstedt, Inc., Newton, NC) was used for reagent and sample preparation to avoid metal contamination. Quality control samples (Utak Laboratories, Inc., Valencia, CA) were used to verify method performance and confirm obtained values were within recommended ranges. Quantitative analyses were performed using the scanning mode of data acquisition. For each element, peak area (signal) was divided (normalized) by the signal from the internal standard. Based on triplicate analyses, the estimated average interassay CV for copper, iron, selenium, and zinc was in the range of 2–7%.

### 2.7. Data Analysis

For all measures, descriptive statistics were calculated and graphs drawn to identify outliers; no data points were determined to be outliers. Target sample size was calculated to include 15 participants per group to detect a minimum difference of 12% in whole blood glutathione with 80% power based on our previous study [15]. Our primary groups of comparisons were as follows: low dose FDS versus high dose FDS, and low and high dose FDS versus calorie- and fiber-matched control groups. For each variable, mean differences between strawberry and control groups at baseline and at 12 weeks were assessed using the multivariate analysis of variance (MANOVA), followed by Bonferroni* post hoc* analyses. All statistical tests were 2-tailed with significance level set at 0.05. SPSS for Windows (version 15.0, SPSS, 2006) was used for the statistical calculations.

## 3. Results

**Figure 1:**
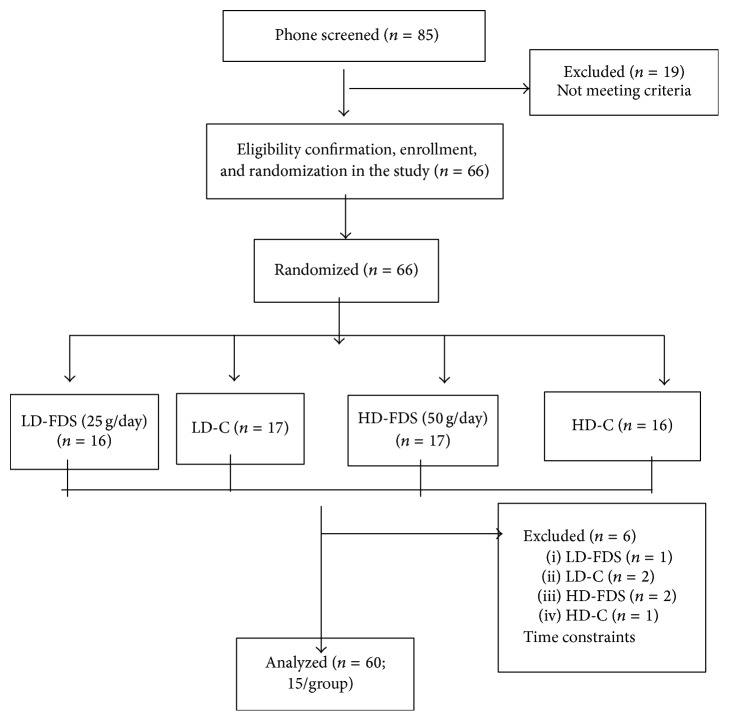
Flow of participants through the trial. C, control; FDS, freeze-dried strawberries; HD, high dose; LD, low dose.

The baseline characteristics of our study participants are presented in Table 2. No significant differences in baseline characteristics were observed among the strawberry groups and the corresponding control groups. As shown in Figure 1, a total of 85 participants were screened for the study, and 66 were enrolled upon satisfying the inclusion and exclusion criteria. Among those enrolled, six participants withdrew because of time constraints and thus, 60 participants completed the 12-wk study in the strawberry and control arms. Among these participants, compliance was 100% for the strawberry groups and 97% for the control groups, as assessed by mandatory weekly visits (3 d/wk) and return of any unconsumed beverages on the days the participants did not come to the clinic. Ellagic acid was detectable in the strawberry group at 12 weeks, but not at baseline as well as in the control groups as reported previously [19]. No adverse events were reported in the study.

### 3.1. Plasma Antioxidant Capacity and Enzyme Activity

Plasma antioxidant capacity and whole blood glutathione were not statistically different at baseline, but were significantly higher in the low and high dose strawberry groups compared to their calorie- and fiber-matched controls (all *p* < 0.01; Table 3). While plasma antioxidant capacity did not differ between the low and high dose strawberry groups, blood glutathione was significantly higher in the high dose versus low dose strawberry group at 12 weeks of the study (*p* < 0.05; Table 3). Serum catalase activity tended to be lower in the high dose strawberry when compared to the calorie- and fiber-matched controls (*p* = 0.06), while catalase activity was significantly higher in the low dose strawberry versus low dose calorie- and fiber-matched controls at 12 weeks (*p* < 0.01; Table 3). No differences were noted in glutathione peroxidase and glutathione reductase enzyme activities among any groups at baseline or at 12 weeks of the study (Table 3).

### 3.2. Plasma Trace Elements and Micronutrient Intakes

We did not observe any differences in plasma copper, iron, selenium, and zinc among any groups at baseline and at 12 weeks of the study (Table 3). Dietary intakes of selected micronutrients, including trace elements, did not significantly vary among groups at any time point (Table 4).

## 4. Discussion

In this 12-week clinical study, we examined the dose-response effects of dietary strawberries on selected biomarkers of antioxidant status in obese adults with elevated serum lipids. Overall, our findings reveal strawberry intervention to increase plasma antioxidant capacity and whole blood glutathione when compared to the matched controls; the higher dose was associated with a greater increase in glutathione when compared to the lower dose of strawberries. Serum catalase activity was increased only in the low dose strawberry group compared to the controls, while no effects of either dose were noted on glutathione peroxidase and glutathione reductase enzyme activities. Plasma copper, iron, selenium and zinc were also not affected by the strawberry intervention. To our knowledge, this is the first clinical study to report the effects of two achievable dietary doses of strawberries on circulating antioxidant biomarkers in adults with CVD risk factors. These findings may further explain the protective associations of berry fruit intake against CVD risk factors, especially obesity and diabetes as observed in prospective cohort studies [2, 23].

Plasma antioxidant capacity, a biomarker of enzymatic as well as nonenzymatic antioxidants has been shown to be increased by many studies using strawberries and other berry polyphenols [1]. In healthy volunteers, strawberry intervention at doses comparable to those used in our study showed a significant increase in plasma antioxidant capacity indicating a direct absorption of strawberry antioxidants such as polyphenols and vitamins and/or an enhanced production of endogenous antioxidants [24–26]. Among the endogenous antioxidant defense mechanisms, catalase and the glutathione enzyme system play key roles in neutralizing oxidative damage induced by hydrogen peroxide and its subsequent ability to generate the hydroxyl radical [4]. Using animal models of oxidative stress, berry polyphenols have been shown to upregulate the synthesis of intracellular glutathione and glutathione peroxidase activity and attenuate mitochondrial oxidative stress [27]. These mechanistic findings have been supported by a few clinical studies reporting the effects of berries on antioxidant enzymes. A six-week dietary intervention study in junior athletes who were administered an acai berry-based juice blend showed a trend towards increased catalase and glutathione reductase activities, but no effects on glutathione peroxidase at one-hour after exercise phase. However, no effects were noted in enzyme activities in the resting state in these young athletes [28]. Another three-week intervention of an antioxidant rich berry juice blend in hemodialysis patients revealed a significant increase in blood glutathione levels, thus improving oxidative stress in people with chronic kidney disease [29]. In a two-week intervention study in healthy volunteers, a polyphenol-rich juice was also shown to upregulate leucocyte protein expression of the phase II anticarcinogenic and antioxidant enzyme glutathione-S-transferase [30]. In our study, we observed a dose-dependent increase in blood glutathione in our participants with CVD risk factors and a higher activity of catalase enzyme only in the low dose strawberry group. Interestingly, we also observed a trend towards decreased catalase activity following high dose strawberry intervention, similar to a decrease in erythrocyte catalase activity observed after an intervention with high polyphenol orange juice [31]. These effects have been explained by the ability of polyphenols to “spare” endogenous antioxidants and/or regenerate other antioxidants to improve their synergistic action in vivo [31]. In general, whole strawberries contain vitamins and carotenoids as antioxidants in addition to polyphenols [32], and these may modulate endogenous enzyme activities depending on the “baseline antioxidant status” in our participants. While we did not measure plasma vitamins in our study, we report low dietary intakes of vitamin C and E, as well as of fruits and vegetables in our participants when compared to the dietary recommendations [33, 34]. Nevertheless, in view of the fact that more than 80% of the US population do not meet the national recommendations of intakes for fruits and vegetables [34], our study reveals supplementation of low as well as high dose strawberries confer some antioxidant protection in obese adults.

Some trace elements, such as copper and iron, have been shown to increase oxidative stress, while others such as zinc and selenium are essential constituents of the antioxidant enzymes, especially superoxide dismutase and the glutathione enzyme system [4, 6, 35]. Polyphenols have been shown to chelate metal ions including iron and have been proposed as treatment agents in iron overload conditions such as thalassemia [36]. Grape seed polyphenol supplementation in healthy piglets were shown to cause a slight decrease in liver concentrations of copper and zinc, though both elements were within the normal physiological limits when compared to control animals [37]. Clinical studies are limited and show somewhat conflicting results. Cereal grain polyphenols have been shown to decrease postprandial zinc absorption in young adults [38], while a three-month supplementation of green tea polyphenols was reported to increase serum zinc and lower serum iron in obese adults [39]. We have previously reported the effects of green tea polyphenols in lowering plasma iron in obese adults with the metabolic syndrome [15]. In our current study, dietary strawberries at low and high doses had no effects on plasma trace elements in obese adults with elevated serum lipids. Thus, further studies are needed on the role of polyphenols derived from different dietary sources in modulating trace element status in adults with CVD risk factors.

Our study has some limitations including a small sample size and the selection of otherwise healthy obese adults with above optimal serum lipids, and these may explain some of our null findings. Also, while we previously measured lipid peroxidation which revealed a decrease with strawberry supplementation [19], other biomarkers of oxidative stress, such as protein carbonyls and biomarkers of DNA damage, must also be determined in future studies. In addition, we did not measure other biomarkers of antioxidant status, such as circulating or tissue vitamin C, which also play an important role in the cellular antioxidant defense mechanism. Finally, we measured levels of these antioxidants in the fasting or “resting state” following the strawberry intervention; but as shown in previous studies, stress-inducing conditions, such as exercise or the postprandial phase, may elicit a more pronounced difference in these measures of antioxidant enzymes.

## 5. Conclusions

Our study findings support the hypothesis that dietary strawberries selectively increase antioxidant biomarkers in obese adults with elevated serum lipids, especially plasma antioxidant capacity, glutathione, and catalase enzyme activity. Obesity is an underlying risk factor for many chronic conditions, including CVD, and has been associated with increased oxidative stress and antioxidant deficiencies. Thus, dietary strawberries, in addition to providing a significant source of antioxidant polyphenols and vitamin C, can also increase endogenous antioxidant capacity. This may offer additional protection against obesity-related conditions, such as CVD, the metabolic syndrome, and type 2 diabetes.

## Figures and Tables

**Table 1:** Composition of strawberry and control beverages.

Composition	LD-FDS	LD-C	HD-FDS	HD-C
FDS, g	25	—	50	—
Fiber, g	4.0	4.0	8.0	8.0
Calories, kcal	75	80	150	144
Protein, g	1.8	—	3.5	—
Fat, g	0.3	—	0.5	—
Carbohydrates, g	16	20	32	36
Ash, g	1.5	—	3.2	—
Vitamin C, mg	55	—	109	—
Total phenolics^1^, mg	1001	—	2005	—
Total anthocyanins^2^, mg	78	—	155	—
Ellagic acid, mg	106	—	220	—
Phytosterols, mg	23	—	50	—

C, control; FDS, freeze-dried strawberries; HD, high dose; LD, low dose.

^1^Expressed as mg gallic acid equivalents.

^2^Expressed as mg cyanidin-3-glucoside equivalents.

**Table 2:** Baseline characteristics and dietary trace element intake of the study participants^1^.

Variable	LD-FDS	LD-C	HD-FDS	HD-C
	(*n* = 15)	(*n* = 15)	(*n* = 15)	(*n* = 15)
Age (years)	50 ± 10	48 ± 10	49 ± 11	48 ± 10
M/F (*n*/*n*)	1/14	1/14	2/13	1/14
Waist (cm)	104 ± 7.6	109 ± 8.1	114 ± 12.7	107 ± 6.6
Height (cm)	165 ± 5.8	165 ± 6.7	164 ± 6.5	169 ± 7.6
Weight (kg)	95 ± 14	100 ± 12	101 ± 18	99 ± 15
BMI (kg/m^2^)	34.5 ± 4.4	37.0 ± 4.4	38.0 ± 7.1	35.0 ± 5.2
Fasting glucose (mmol/L)	5.5 ± 0.14	5.7 ± 0.18	5.2 ± 0.19	5.3 ± 0.25
Triglycerides (mmol/L)	1.7 ± 0.23	1.4 ± 0.17	1.9 ± 0.20	2.0 ± 0.21
Total cholesterol (mmol/L)	5.2 ± 0.28	5.2 ± 0.13	5.5 ± 0.18	5.3 ± 0.23
LDL-cholesterol (mmol/L)	3.1 ± 0.23	3.1 ± 0.15	3.4 ± 0.18	3.2 ± 0.21
HDL-cholesterol (mmol/L)	1.2 ± 0.10	1.3 ± 0.08	1.3 ± 0.10	1.2 ± 0.08
BUN (mg/dL)	14 ± 2.3	16 ± 4.5	16 ± 4.2	17 ± 5.0
Creatinine (mg/dL)	0.8 ± 0.2	0.8 ± 0.2	0.8 ± 0.2	0.9 ± 0.3
AST (U/L)	29 ± 10	26 ± 7	25 ± 4	25 ± 7
ALT (U/L)	34 ± 12	34 ± 12	30 ± 11	31 ± 12
WBC (*n* × 10^−3^)	6.6 ± 1.2	6.7 ± 1.4	6.7 ± 1.7	7.1 ± 1.5
RBC (*n* × 10^−6^)	4.6 ± 0.5	4.5 ± 0.3	4.6 ± 0.6	4.8 ± 0.4
Hb (g/dL)	14 ± 1.4	14 ± 1.3	14 ± 1.4	14 ± 1.6
Multivitamin users (%)	20.0	20.0	20.0	10.0
Fruit servings (*n*/wk)	1.2	1.0	1.2	1.0
Vegetable servings (*n*/wk)	1.0	1.1	1.1	1.2

^1^Values are mean ± SD.

AST, aspartate aminotransferase; ALT, alanine aminotransferase; BMI, body mass index; BUN, blood urea nitrogen; C, control; FDS, freeze-dried strawberries; Hb, hemoglobin; HD, high dose; LD, low dose; RBC, red blood cell; WBC, white blood cell.

No significant differences were noted among any groups using the multivariate analysis of variance (MANOVA) at baseline for each variable.

**Table 3:** Antioxidant enzyme activities and trace element status following a 12-week strawberry or placebo beverage supplementation.

Variable	LD-FDS	LD-C	HD-FDS	HD-C
	(*n* = 15)	(*n* = 15)	(*n* = 15)	(*n* = 15)
Plasma antioxidant capacity (*µ*mol/L)				
0 wk	1.6 ± 0.2	1.7 ± 0.3	1.8 ± 0.3	1.5 ± 0.3
12 wk	2.9 ± 0.3^*∗*^	1.6 ± 0.3	3.1 ± 0.3^*∗*^	1.2 ± 0.2
Serum catalase (U/mL)				
0 wk	38 ± 12	47 ± 11	52 ± 15	45 ± 20
12 wk	67 ± 23^*∗*^	32 ± 9	40 ± 13^#^	54 ± 18
Serum glutathione peroxidase (mU/mL)				
0 wk	13.9 ± 4.0	13.7 ± 3.8	14.7 ± 4.5	12.4 ± 2.8
12 wk	13.2 ± 3.9	14.0 ± 4.7	14.6 ± 3.3	11.5 ± 2.0
Serum glutathione reductase (U/L)				
0 wk	35.7 ± 17	30.5 ± 12	36.1 ± 9	40.4 ± 18
12 wk	45.1 ± 21	28.5 ± 17	32.6 ± 12	37.2 ± 21
Whole blood glutathione (*µ*g/g Hb)				
0 wk	1657 ± 57	1898 ± 52	1824 ± 38	1789 ± 63
12 wk	2295 ± 158^*∗*,¶^	1775 ± 48	2860 ± 121^*∗*^	1862 ± 62
Plasma iron (mg/dL)				
0 wk	0.9 ± 0.1	1.8 ± 0.6	1.0 ± 0.1	0.9 ± 0.1
12 wk	1.2 ± 0.1	1.0 ± 0.1	1.1 ± 0.2	1.3 ± 0.3
Plasma copper (mg/dL)				
0 wk	1.3 ± 0.1	1.4 ± 0.2	1.8 ± 0.4	1.3 ± 0.1
12 wk	1.5 ± 0.1	1.6 ± 0.1	1.4 ± 0.1	1.5 ± 0.1
Plasma selenium (mg/dL)				
0 wk	0.10 ± 0.02	0.14 ± 0.03	0.15 ± 0.02	0.13 ± 0.02
12 wk	0.09 ± 0.02	0.11 ± 0.03	0.14 ± 0.02	0.11 ± 0.03
Plasma zinc (mg/dL)				
0 wk	1.1 ± 0.2	1.3 ± 0.3	1.2 ± 0.1	1.0 ± 0.2
12 wk	0.6 ± 0.1	0.9 ± 0.2	0.9 ± 0.2	1.0 ± 0.2

Values are mean ± SE.

C, control; FDS, freeze-dried strawberries; Hb, hemoglobin; HD, high dose; LD, low dose; RBC, red blood cell; WBC, white blood cell.

*p* values are derived from the multivariate analysis of variance (MANOVA) assessing differences between strawberry and control groups at 0 and 12 wk of the study for each variable.

^*∗*^ *p* < 0.01 versus respective controls; ^¶^ *p* < 0.01 versus HD-FDS; ^#^ *p* = 0.06 versus HD-C.

**Table 4:** Dietary micronutrient intake at baseline and 12 weeks of the study.

Variable	LD-FDS	LD-C	HD-FDS	HD-C
	(*n* = 15)	(*n* = 15)	(*n* = 15)	(*n* = 15)
Copper intake (mg/d)				
0 wk	0.7 ± 0.3	0.6 ± 0.3	0.7 ± 0.2	0.65 ± 0.3
12 wk	0.8 ± 0.3	0.6 ± 0.3	0.6 ± 0.2	0.8 ± 0.3
Iron intake (mg/d)				
0 wk	10 ± 8	12 ± 10	15 ± 7	9 ± 7
12 wk	12 ± 8	13 ± 9	13 ± 10	11 ± 9
Selenium intake (*µ*g/d)				
0 wk	37 ± 15	45 ± 22	30 ± 12	38 ± 15
12 wk	41 ± 13	47 ± 20	35 ± 10	40 ± 10
Zinc intake (mg/d)				
0 wk	6.8 ± 4.3	7.3 ± 5.5	7.1 ± 3.3	8.5 ± 4.0
12 wk	7.2 ± 3.5	8.1 ± 4.5	7.8 ± 2.7	9.3 ± 3.5
Vitamin C intake (mg/d)				
0 wk	40 ± 5.0	39 ± 7.6	35 ± 3.7	35 ± 3.8
12 wk	37 ± 8.9	34 ± 4.6	38 ± 5.5	31 ± 2.8
Vitamin E intake (mg/d)				
0 wk	1.3 ± 0.2	1.2 ± 0.2	1.2 ± 0.2	1.4 ± 0.2
12 wk	1.5 ± 0.3	1.0 ± 0.1	1.7 ± 0.3	1.2 ± 0.1
Beta-carotene intake (mg/d)				
0 wk	2.5 ± 0.8	2.1 ± 0.5	1.8 ± 0.6	2.0 ± 0.7
12 wk	1.8 ± 0.9	2.4 ± 0.8	2.1 ± 0.8	1.7 ± 0.5

Values are mean ± SE.

C, control; FDS, freeze-dried strawberries; HD, high dose; LD, low dose.

No significant differences were noted among any groups using the multivariate analysis of variance (MANOVA) at baseline and 12 wk for each variable.
